# P-2212. Nasal microbiota differences in young infants and early childhood neurobehavior: a pilot study

**DOI:** 10.1093/ofid/ofae631.2366

**Published:** 2025-01-29

**Authors:** Leena B Mithal, Aspen N Kremer, Aspen N Kremer, Laura Shanahan, Stefan J Green, Ankur Naqib, Mary Akel, Yudong Zhang, Emma Office, Sebastian Otero, Marci Messina, Lawrence Gray, Elizabeth Norton, Thorsten Kahnt, Lauren Wakschlag, Patrick C Seed

**Affiliations:** Ann and Robert H. Lurie Children's Hospital of Chicago, Chicago, Illinois; Ann & Robert H. Lurie Children's Hospital, Chicago, Illinois; Ann & Robert H. Lurie Children's Hospital, Chicago, Illinois; Rhodes College, Memphis, Tennessee; Rush University Medical Center, Chicago, Illinois; Rush University Medical Center, Chicago, Illinois; Lurie Children's Hospital, Chicago, Illinois; Northwestern University, Chicago, Illinois; University of Chicago, Chicago, Illinois; ECHO Chicago, Chicago, Illinois; Northwestern Medicine, Chicago, Illinois; Lurie Children's Hospital, Chicago, Illinois; Northwestern University, Chicago, Illinois; National Institutes of Health, Baltimore, Maryland; Northwestern University, Chicago, Illinois; Lurie Children's Hospital/Northwestern University Feinberg School of Medicine, Chicago, Illinois

## Abstract

**Background:**

The etiology and mediators of childhood neurobehavioral conditions are poorly understood. The early life microbiome is implicated in neurodevelopment. In contrast to gut microbiota, however, the relationship between nasal microbiota and early behavior remains unknown. Olfaction serves critical functions in neonates. Murine data suggests that nasal inoculation of *E. coli* in neonates drives different adolescent behavior outcomes. We hypothesized that the newborn nasal microbiota influences olfactory perception and early life behavioral development.

Study design and data acquisition

**Figure:**
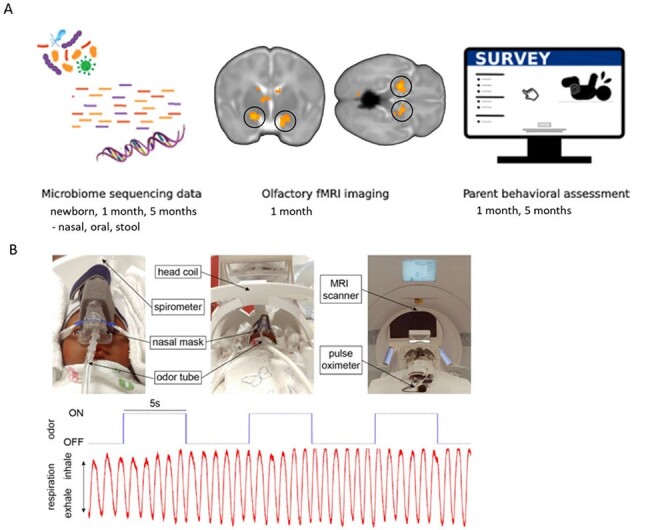

a) Microbiota data from nasal, oral, and fecal samples at newborn, 1 month, and 5 month timepoints; olfactory fMRI brain imaging at 1 month; and parent neurobehavioral assessment at 1 and 5 months. b) Infant olfactory MRI setup and respiration trace during odor delivery via spirometer on nasal mask.

**Methods:**

In this prospective pilot study, thirteen vaginally-delivered infants were enrolled. Nasal, oral, and fecal microbiome specimens were collected at three time points: newborn (1-2 days), 1 month, and 5 months of age. Neurobehavioral assessments were performed at 1 and 5 months. At 1 month, infants had an olfactory brain fMRI scan in a natural sleep state during which pleasant and unpleasant odors were delivered via nasal mask (Fig 1). Extracted biospecimen DNA was subjected to 16S rRNA next generation sequencing (V1-V3 region, 27F-534R primers). Bioinformatics analysis of diversity, differential microbial features, and correlations were conducted in R.

Nasal microbiota profiles for infants

**Figure:**
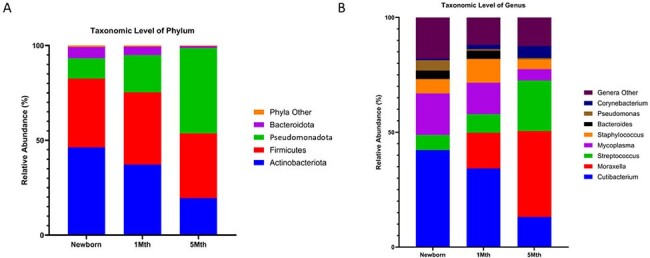

16S rRNA next generation sequencing of nasal samples of patients (n=13) at newborn, 1 month, and 5 month time points at the a) taxonomic level of phylum and b) taxonomic level of genus with top ten taxa shown.

**Results:**

Alpha-diversity of nasal microbiota did not vary by age. Beta-diversity differences were observed between newborn and 1 month (PERMANOVA; p = 0.0521) and newborn and 5 months (p = 0.0027). At the taxonomic level of phylum, the relative abundance of Actinomycetota and Bacteroidota was significantly lower and that of Pseudomonadota significantly higher at 5 months compared to newborns. At the genus level, the relative abundance of Moraxella increased from newborn to 5 months (p< 0.0001), and Streptococcus relative abundance was significantly higher at 5 months (Fig 2). Infants with high irritability (n=4, Fig 3) demonstrated nasal microbiota differences compared to those without (n=9) at newborn, 1 month, and 5 month time points. Analysis of odor-evoked cortical activation on fMRI and toddler behavioral assessments are in process.

Infant behavioral dysregulation scores

**Figure:**
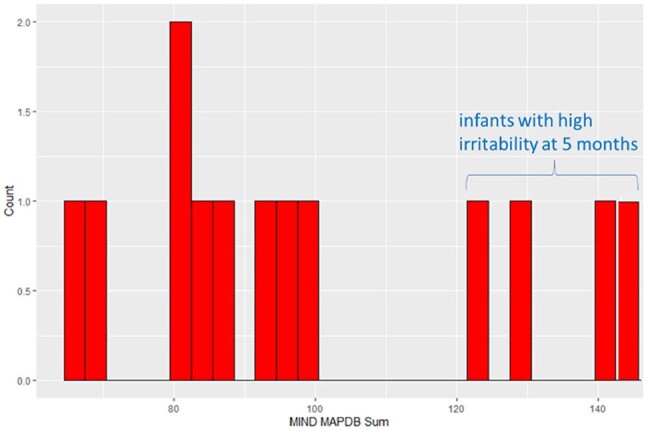

Distribution of the Multidimensional Assessment Profiles Temper Loss (MAPS-TL) Scales scores for n=13 infants at 5-6 months. X-axis is the numerical MAPS-TL score and y-axis is number of infants. Infants with high irritability (n=4) cluster with higher scores.

**Conclusion:**

Nasal microbiota differences in early infancy are associated with behavioral dysregulation. Further investigation of how nasal microbiota may impact neurobehavioral health, potentially via olfaction, is warranted.

**Disclosures:**

All Authors: No reported disclosures

